# Supplementary material for: Strong lowering of ionization energy of metallic clusters by organic ligands without changing shell filling
Source: Nat Commun. 2018 Jun 15;9:2357. doi: 10.1038/s41467-018-04799-0 (PMC6003947; doi:10.1038/s41467-018-04799-0)
Supplement: Supplementary file 1 — Supplementary Information [file 41467_2018_4799_MOESM1_ESM.pdf]

Strong Lowering of Ionization Energy of Metallic Clusters by Organic Ligands Without Changing Shell Filling

Chauhan et al.

Department of Physics

Virginia Commonwealth University,

Richmond, VA, 23284-2000, USA

Cluster	AIP	HOMO	LUMO	Gap
	eV	eV	eV	eV
Al_{13}	6.13	-5.108	-4.700	0.408
BAl_{12}	6.64	-5.101	-4.838	0.263
CAl_{12}	6.63	-5.127	-3.075	2.052
PAl_{12}	5.10	-3.724	-3.156	0.568
$SiAl_{12}$	6.73	-5.168	-3.182	1.986
EP+Al_{13}	5.08	-4.407	-3.697	0.710
EP+BAl_{12}	5.57	-4.204	-3.588	0.616
EP+CAl_{12}	5.57	-4.254	-2.431	1.823
EP+PAl_{12}	4.36	-3.134	-2.517	0.617
EP+$SiAl_{12}$	5.73	-4.445	-2.573	1.872
2EP+Al_{13}	4.35	-3.703	-3.166	0.530
2EP+BAl_{12}	4.62	-3.531	-2.986	0.545
2EP+CAl_{12}	4.77	-3.573	-1.867	1.706
2EP+PAl_{12}	3.69	-2.592	-1.974	0.617
2EP+$SiAl_{12}$	4.97	-3.784	-2.033	1.751
3EP+Al_{13}	3.51	-2.689	-2.330	0.358
3EP+BAl_{12}	3.68	-2.941	-2.463	0.478
3EP+CAl_{12}	4.16	-3.028	-1.469	1.559
3EP+PAl_{12}	3.25	-2.305	-1.747	0.558
3EP+$SiAl_{12}$	4.41	-3.321	-1.727	1.594

Supplementary Table 1. Adiabatic ionization energy (AIE), the energies of HOMO, LUMO, and HOMO-LUMO gap of the $Al_{13}(EP)_n$ and $MAl_{12}(EP)_n$ (n=0-3) clusters where M=C, Si, B, and P.

Mulliken Population Analysis				
	1EP	2EP	3EP	Average
CAl_{12}	-0.38	-0.61	-0.88	-0.29
$SiAl_{12}$	-0.36	-0.59	-0.86	-0.29
BAl_{12}	-0.40	-0.62	-0.88	-0.29
Al_{13}	-0.39	-0.63	-0.90	-0.30
PAl_{12}	-0.35	-0.56	-0.83	-0.28

Supplementary Table 2. Mulliken population analysis of $Al_{13}(EP)_n$ and $MAl_{12}(EP)_n$ (n=0-3) Clusters where M=C, Si, B, and P.

	Moment	Total Energy	BE
EP	0	-110.15527118	
CAl_{12}	0	-41.43146917	
$CAl_{12}(EP)$	0	-152.35413142	0.76739107
$CAl_{12}(EP)_2$	0	-263.21690749	0.70750489
$CAl_{12}(EP)_3$	0	-373.88539069	0.51321202
BAl_{12}	1	-39.49094048	
$BAl_{12}(EP)$	1	-150.78015034	1.13393868
$BAl_{12}(EP)_2$	1	-261.62601711	0.69059559
$BAl_{12}(EP)_3$	1	-372.25486334	0.47357505
PAl12	1	-39.36669131	
$PAl_{12}(EP)$	1	-150.36612463	0.84416214
$PAl_{12}(EP)_2$	1	-261.2891261	0.76773029
$PAl_{12}(EP)_3$	1	-372.05475141	0.61035413
SiAl12	0	-39.65755298	
$SiAl_{12}(EP)$	0	-150.55764815	0.74482399
$SiAl_{12}(EP)_2$	0	-261.42262543	0.7097061
$SiAl_{12}(EP)_3$	0	-372.07769539	0.49979878
Al_{13}	1	-36.53766447	
$Al_{13}(EP)$	1	-147.74465724	1.05172159
$Al_{13}(EP)_2$	1	-258.59494303	0.69501461
$Al_{13}(EP)_3$	1	-369.23666932	0.48645511

Supplementary Table 3. Successive ligand binding energy in $Al_{13}(EP)_n$ and $MAl_{12}(EP)_n$ (n=0-3) clusters where M=C, Si, B, and P.

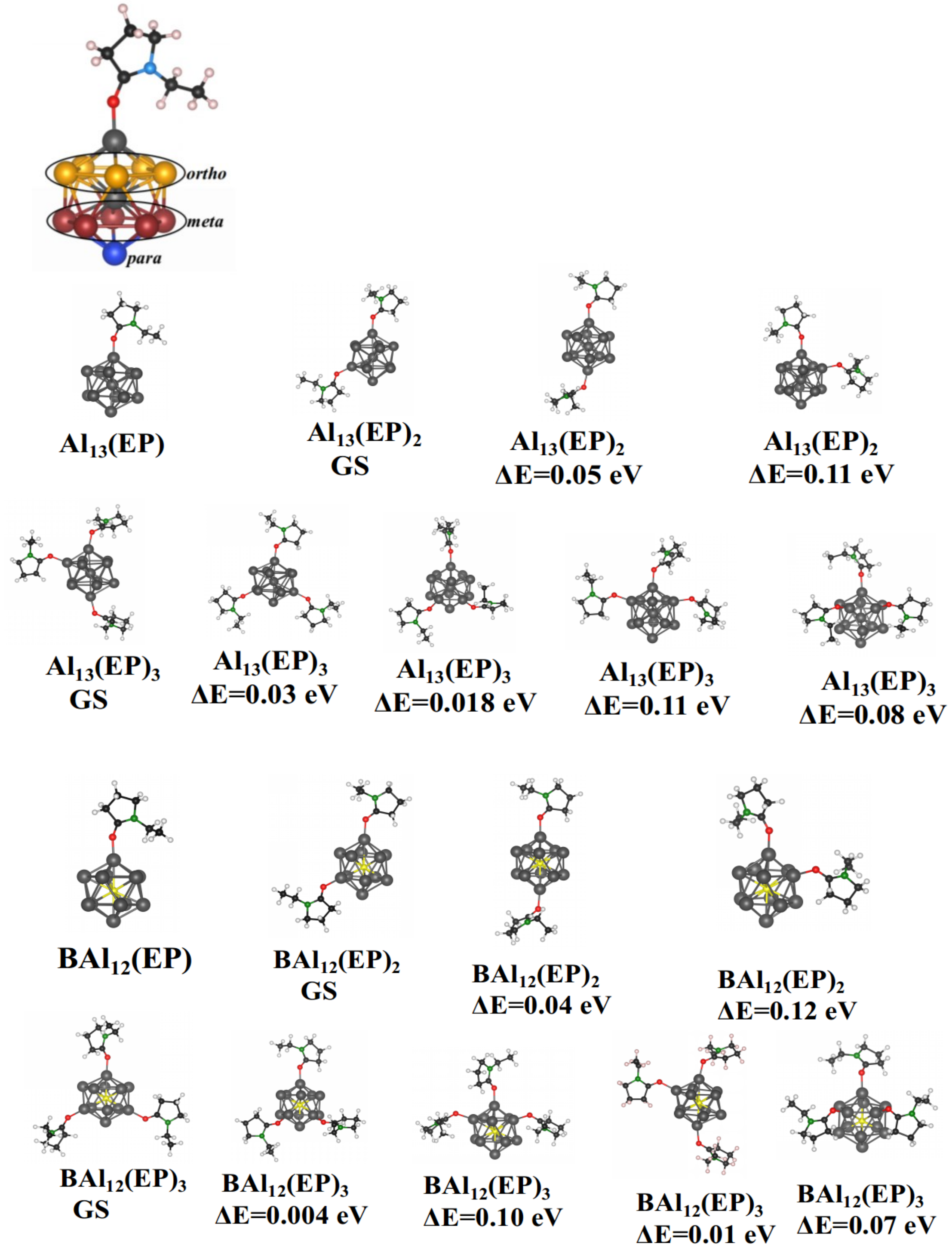

Supplementary Figure 1. Ground states structures along with higher energy isomers of Al_{13}, BAl_{12} clusters, and their ligated derivatives with EP ligands. The *ortho, meta* and *para* positions relative to 1st EP ligands are also shown.

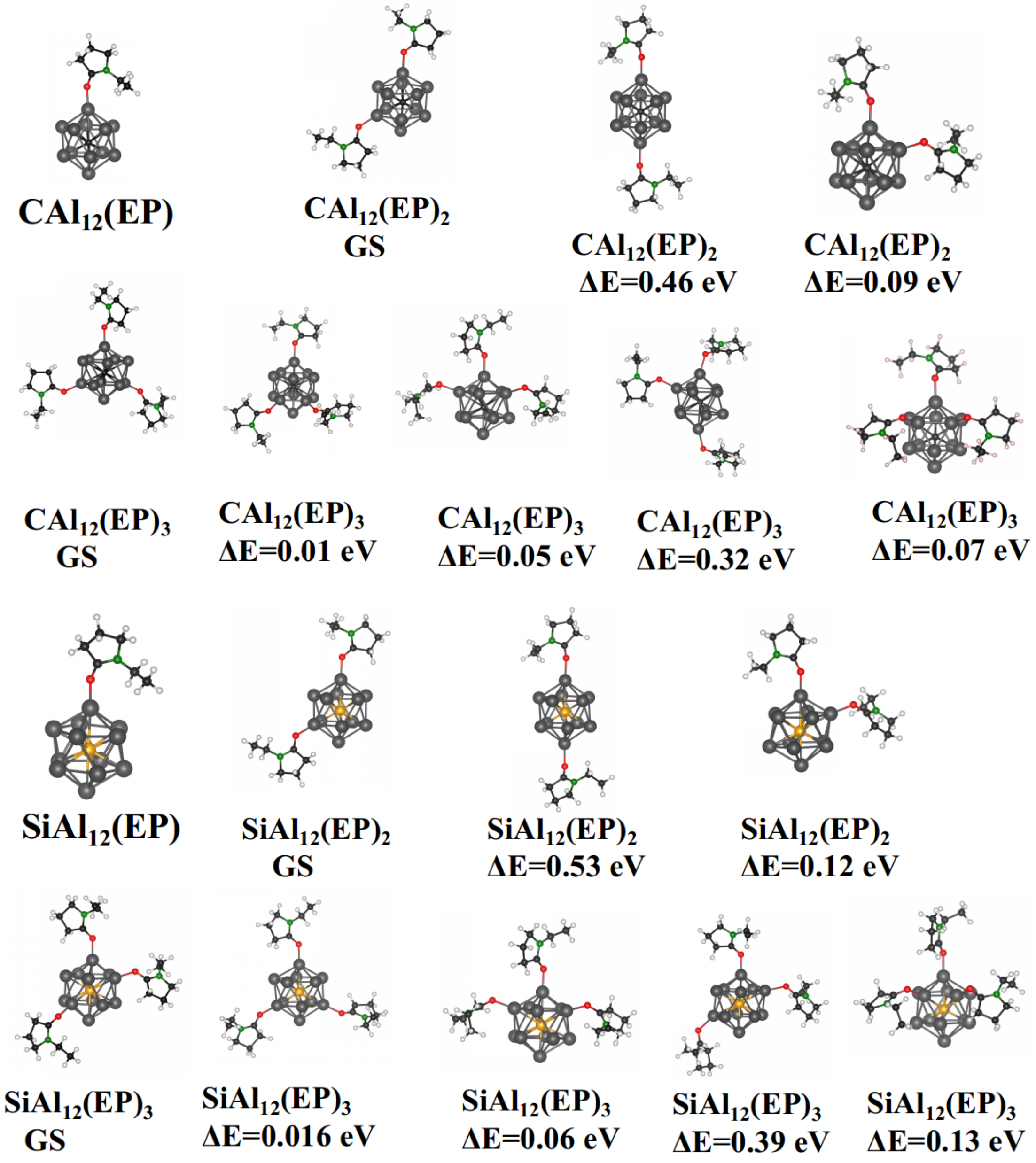

Supplementary Figure 2. Ground states structures along with higher energy isomers of CAl_{12}, $SiAl_{12}$ clusters, and their ligated derivatives with EP ligands.

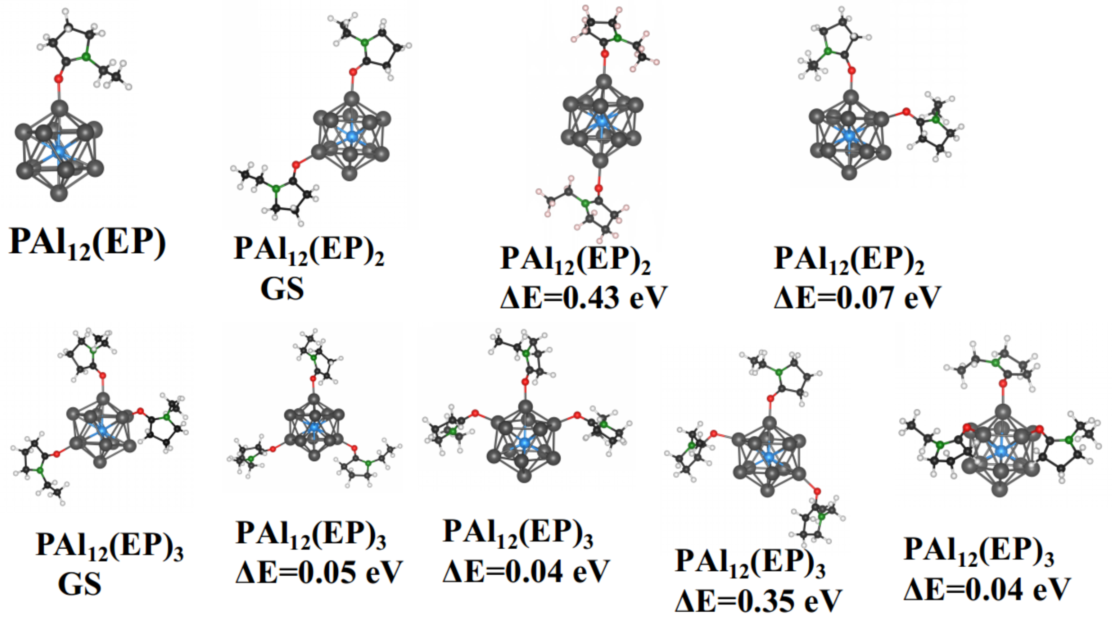

Supplementary Figure 3. Ground states structures along with higher energy isomers of PAl_{12} clusters and their ligated derivatives with EP ligands.

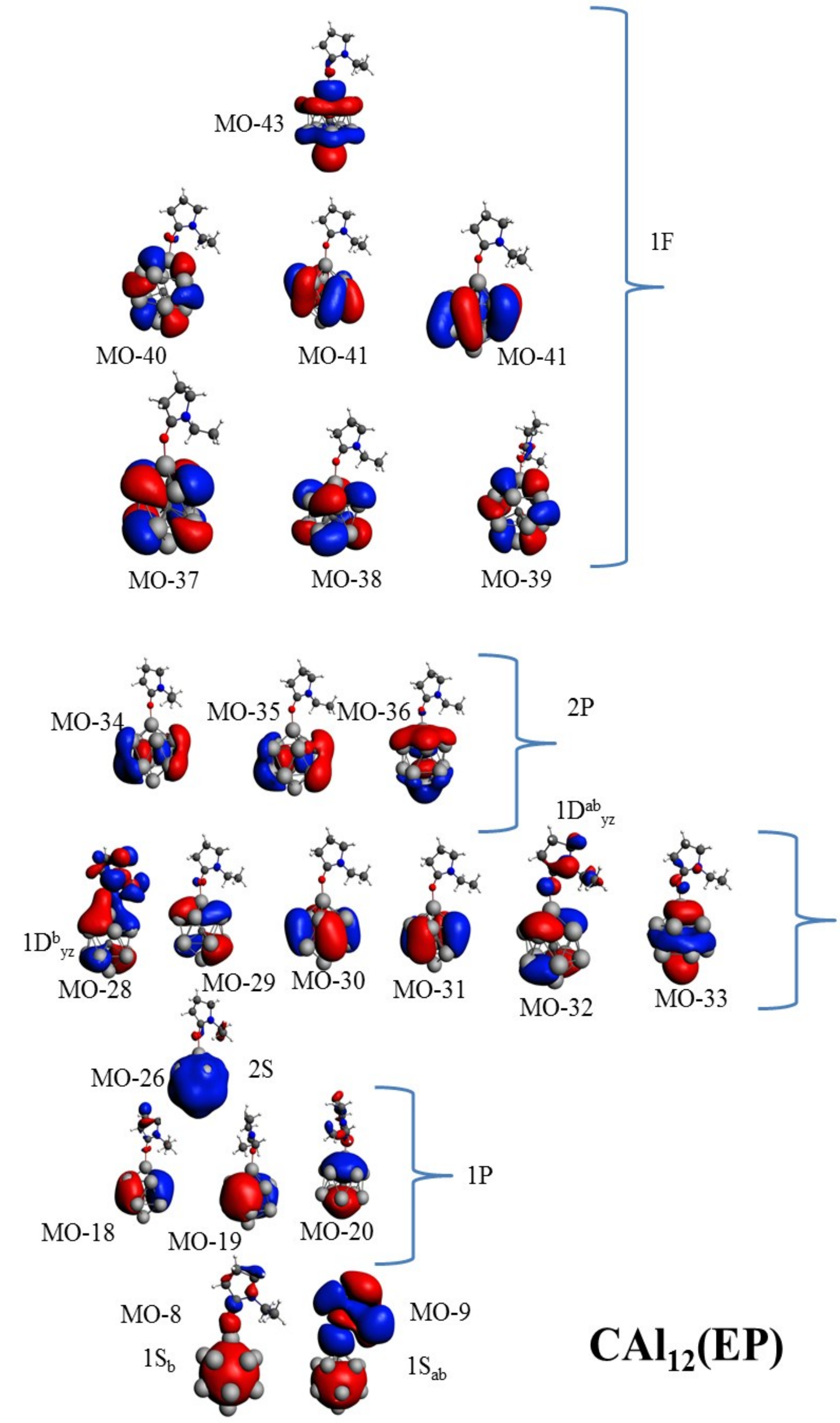

Supplementary Figure 4. Molecular orbital iso-surfaces of CAl_{12}(EP) cluster marked with their angular characters.

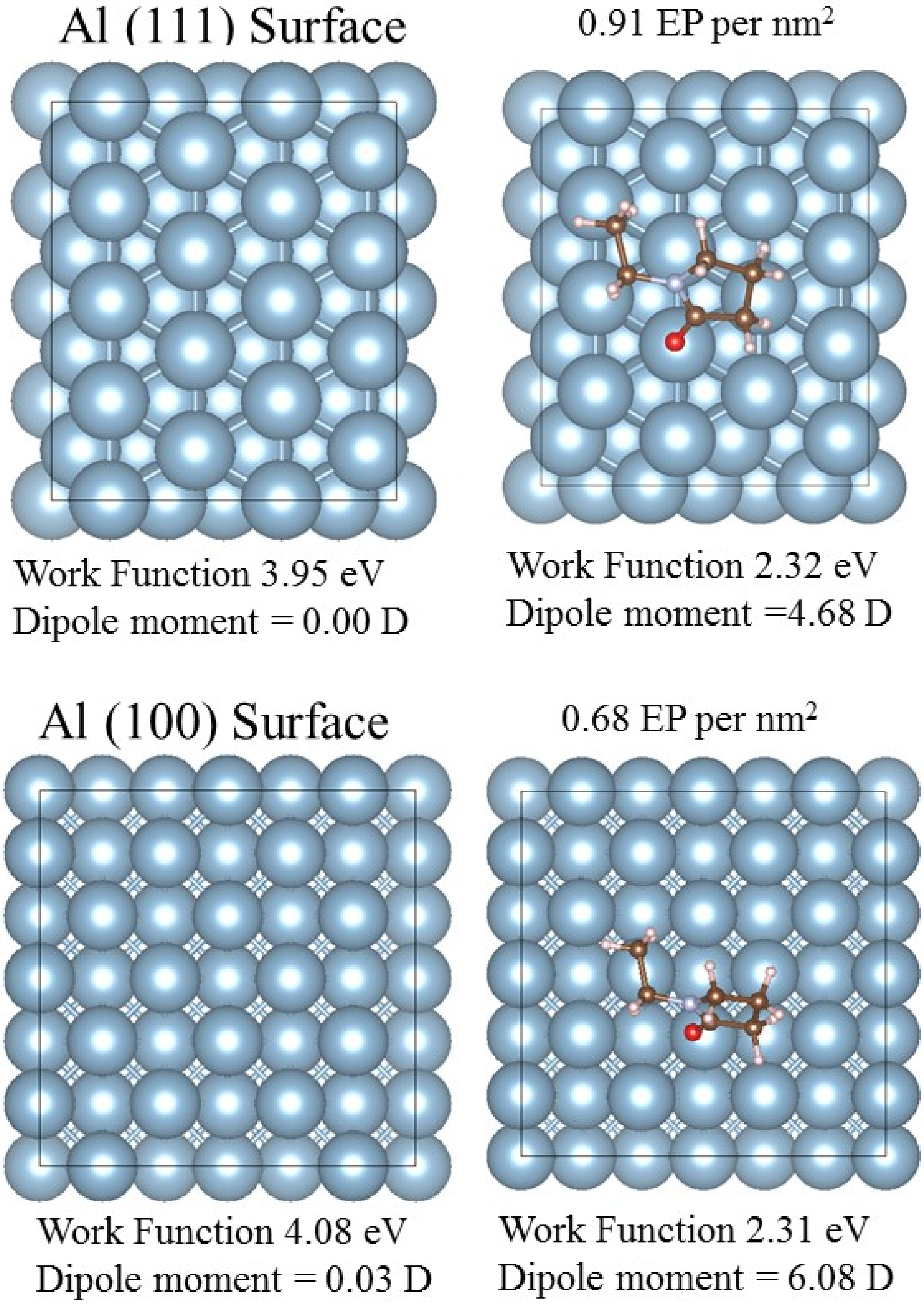

Supplementary Figure 5. The calculated work function of Al (111) and Al (100) surfaces before and after the binding of an EP molecule. The dipole moment perpendicular to the surface in Debye, and the EP coverage per square nanometer are shown.
